# Determinants and patterns of care-seeking for childhood illness in rural Pune District, India

**DOI:** 10.7189/jogh.10.010601

**Published:** 2020-06

**Authors:** Andrew Marsh, Siddhivinayak Hirve, Pallavi Lele, Uddhavi Chavan, Tathagata Bhattacharjee, Harish Nair, Sanjay Juvekar, Harry Campbell

**Affiliations:** 1 Institute for International Programs, Johns Hopkins University Bloomberg School of Public Health, Baltimore, Maryland, USA; 2 KEM Hospital Research Centre, Rasta Peth, Pune, India; 3 INDEPTH Network, East Legon, Accra, Ghana; 4 Usher Institute of Population Health Sciences and Informatics, University of Edinburgh, Teviot Place, Edinburgh, UK; * Joint last author with equal contributions

## Abstract

**Background:**

An estimated 1.2 million children under five years of age die each year in India, with pneumonia and diarrhea among the leading causes. Increasing care-seeking is important to reduce mortality and morbidity from these causes. This paper explores the determinants and patterns of care-seeking for childhood illness in rural Pune district, India.

**Methods:**

Mothers having at least one child <5 years from the study area of the Vadu Health and Demographic Surveillance System were enrolled in a prospective cohort study. Household sociodemographic information was collected through a baseline questionnaire administered at enrollment. Participants were visited up to six times between July 2015 and February 2016 to collect information on recent childhood acute illness and associated care-seeking behavior. Multivariate logistic regression explored the associations between care-seeking and child, participant, and household characteristics.

**Results:**

We enrolled 743 mothers with 1066 eligible children, completing 2585 follow-up interviews (90% completion). Overall acute illness prevalence in children was 26% with care sought from a health facility during 71% of episodes. Multivariable logistic regression showed care-seeking was associated with the number of reported symptoms (Odds ratio (OR) = 2.4, 95% confidence interval (CI) = 1.5-3.9) and household insurance coverage (OR = 2.2, 95% CI = 1.1-4.3). We observed an interaction between the associations of illness severity and maternal employment on care-seeking. Somewhat-to-very severe illness was associated with increased care-seeking among both employed (OR = 5.0, 95% CI = 2.2-11.1) and currently unemployed mothers (OR = 7.0, 95% CI = 3.9-12.6). Maternal employment was associated with reduced care-seeking for non-severe illness (OR = 0.3, 95% CI = 0.1-0.7), but not associated with care-seeking for somewhat-to-very severe illness. Child sex was not associated with care-seeking.

**Conclusions:**

This study demonstrates the importance of illness characteristics in determining facility-based care-seeking while also suggesting that maternal employment resulted in decreased care-seeking among non-severe illness episodes. The nature of the association between maternal employment and care-seeking is unclear and should be explored through additional studies. Similarly, the absence of male bias in care-seeking should be examined to assess for potential bias at other stages in the management of childhood illness.

An estimated 5.9 million children under five years of age die each year globally with 1.2 million of these deaths occurring in India [1]. Acute respiratory infections (ARI) and diarrhea are among the leading causes of child mortality, yet many of these deaths may be avoided through scaling up coverage of effective interventions [2]. Care-seeking from a qualified health provider is a necessary step for the management of childhood illnesses, especially when appropriate treatment cannot be provided within the home [3]. Care-seeking for childhood illnesses in India has increased in the last decade, yet substantial gaps remain. An estimated 32% of children with diarrhea, 27% of children with fever, and 22% of children with symptoms of ARI were not brought for care in 2015-16, compared to 41%, 30%, and 31% of children with these illnesses in 2005-06 [4,5].

Data on care-seeking for childhood illness and associated determinants are frequently measured through large household surveys, such as the Demographic and Health Survey (DHS), implemented within India as the National Family and Health Survey (NFHS), Multiple Indicator Cluster Surveys, and Malaria Indicator Surveys [6-9]. A meta-analysis of 258 surveys from these three sources administered between 2000 and 2013 identified common factors associated with care-seeking from a health facility, including child age, child sex, maternal education, urban residence, socioeconomic status (SES), and distance from the nearest health facility [10]. Care-seeking has been associated with illness severity, measured either according to caregiver perception or clinical criteria [11]. While medical professionals and policymakers may be most interested in clinically meaningful illness characteristics, evidence suggests that caregivers may be unable to accurately classify illnesses according to these criteria [12]. Furthermore, a multi-country study of care-seeking for childhood illness observed a strong association between the perception of illness and both illness symptoms and the decision to seek care [13].

Recent reviews of diarrheal disease and ARI in India noted variability in care-seeking practices by region, contrasting high care-seeking for childhood illness from predominantly allopathic providers in the southern state of Kerala with the urban city of Lucknow in the northern India, where traditional healers were visited for 24% and 33% of episodes of neonatal ARI and diarrhea, respectively [14-17]. Care-seeking for childhood illness is higher in Maharashtra than in the national average for India with 89% of children with symptoms of ARI and 78% of children with diarrhea brought for care in Maharashtra compared to 78% and 68% of children with the same illnesses nationally [5]. Care is most frequently sought in the private sector, with wealthier households being more than twice as likely to seek care from a private provider [18]. A mixed-methods study of care-seeking for neonatal and childhood illnesses in rural Maharashtra noted that while private sector practitioners were the most common source of care for each condition, faith-based healers were frequently visited for selected neonatal danger signs (poor sucking, difficult breathing, and boils over body) and childhood measles [19].

Male bias has also been associated with care-seeking behavior with an analysis of national data showing increased delays in care-seeking for girls [20]. A study from rural Maharashtra found male bias across various stages of the care-seeking continuum, noting that caregivers of male children were more likely to seek care from a private practitioner, more likely to comply with referral, tended to spend more money on treatment, and were willing to travel further distance compared with caregivers of female children [21]. The most recent state-level data suggest no difference by child sex in facility-based care-seeking for childhood illness [5], though these data do not rule out male bias in provider choice, delays between onset and the decision to seek care, and household expenditures for care.

While large-scale household surveys collect data across a range of topics, their multipurpose approach restricts the detail they may provide on any single topic. For example, the NFHS questionnaire asks mothers of recently ill children whether care was sought, the facility types where care was sought, and which type of facility was accessed first if multiple types are indicated [5]. These data indicate care-seeking from a specific facility type and whether that type was the first contact with the health system, but cannot describe the complete sequence of care-seeking events, including provider shopping behavior or repeated visits to the same provider type as has been explored elsewhere [22-24]. Additionally, studies evaluating the relationship between health facility access and care-seeking typically measure access according to participant-reported travel time or travel distance [25-27], though these may provide less precise measurements of access relative to more sophisticated GIS approaches.

As part of a study examining the validity of maternal recall of care-seeking for childhood illnesses in rural Pune district, India, we explored the determinants and patterns of care-seeking for diarrhea, fever, and cough among a cohort of children under five. Through an expanded questionnaire on care-seeking practices, we built on the approach of common household questionnaires to include data on sequential care-seeking actions, specific providers visited, and maternally-reported illness severity. This paper describes the results of data collected over six months of follow up with the specific objectives of identifying the factors associated with facility-based care-seeking and describing the sequential patterns of care-seeking for childhood illness.

## METHODS

### Study site

We conducted a prospective cohort study of care-seeking for childhood illness among mothers with young children in rural Pune district, India from May 2015 to February 2016. The study took place within the 22 villages of the Vadu Health and Demographic Surveillance System (HDSS), located 30 km northeast of Pune city, Maharashtra state, India [28]. The study area is served by seven public sector health facilities, including one rural hospital, one primary health center, and five sub-centers. The private sector includes 93 private hospitals and clinics and 68 pharmacies [29]. Several non-facility public sector sources in the study area provide limited treatment for childhood illness, such as Anganwadi/Integrated Child Development Services (ICDS) centers and Accredited Social Health Activist (ASHA) workers.

### Participant enrollment and follow-up

Study participants were mothers 15-49 years old with at least one child less than five years old. A total of 926 mothers were randomly sampled from the Vadu HDSS population register to achieve a target sample size of 750 mothers, allowing for 10% of sampled mothers being ineligible to participate and a further 10% of mothers refusing consent. Study recruitment occurred during field worker home visit. Consenting participants were randomly assigned by computer software to one of three study groups according to the objectives of the parent study. Participants assigned to the primary study group (“phone group”) were provided with a GPS-enabled smartphone and followed up at six monthly visits. Participants in the longitudinal comparison group were similarly followed up at six monthly visits but not provided with smartphones. Participants in the cross-sectional comparison group were also not provided phones and were divided into six subgroups, each followed up once over the six-month follow-up period (Figure S1 in the **Online Supplementary Document**). Comparison groups were included to evaluate potential biases in reported care-seeking behavior among the phone group due to the presence of the smartphone or the repeated study contacts (see [29] and [30] for additional details). At each follow-up visit mothers were administered the NFHS module on childhood illness [5], which asks mothers whether their child experienced diarrhea, fever, or cough in the previous two weeks, whether any care was sought, and, if so, the sources from where care was sought. Mothers reporting a child with one or more of these symptoms were administered a supplementary questionnaire on symptom timing and the specific care-seeking actions taken in response, if any.

### Measures

Our analysis of determinants of facility-based care-seeking is defined as any reported care-seeking from a public or private sector hospital or clinic. As in the NFHS definition, this also included care provided by auxiliary nurse midwives at public sector sub-centers and excluded reported care-seeking from pharmacies, traditional healers, and shops. Additionally, we excluded care sought from Anganwadi/ICDS centers and ASHA workers, as these sources are limited in their capacity to provide comprehensive care for childhood illness.

Explanatory variables included characteristics of the illness, all of which were assessed at follow up, and characteristics of the child and household, which were assessed at baseline. Illness characteristics included illness severity (categorized as non-severe, somewhat severe, or very severe as in [13]), number of reported symptoms (diarrhea, fever, or cough alone vs multiple symptoms), and the presence of any danger signs (vomiting, difficulty eating, being unusually sleepy, or convulsions). We classified illness severity according to mothers’ perception rather than through the presence of clinically-defined symptoms, a decision taken in part because recent evidence suggests mothers have low discriminative power when differentiating between clinically meaningful presentations of similar symptoms [12,31]. The perception of severity has been associated with actual severity, though culturally-specific interpretations of signs and symptoms may also lead to concern [13]. We also expect that illness perception contributes more directly to a mother’s decision to seek care than do the underlying clinical symptoms. Child variables included age at follow-up and sex. Household variables included maternal age, education, and employment status, number of children under-five in the household, religion of household head, SES, household structure (nuclear, extended), health insurance coverage, residence (urban, rural), and distance to the nearest health facility.

SES was determined according to the principal components analysis approach used by DHS [32,33]. Input variables included ownership of various assets (property, various durable goods, agricultural land, livestock, a bank/post office account, and health insurance or a health scheme), household building materials, drinking water source, toilet facility, and the presence of a servant or maid within the household. Distance to the nearest health facility was calculated as the shortest road-based distance using the inputs of participant and health facility locations coordinates and the current road network within the network analyst extension with ArcGIS 10 [34].

### Statistical analysis

We explored determinants and patterns of care-seeking in response to individual episodes of childhood illness, excluding records for children completing their fifth birthday prior to follow-up visit. We estimated the unadjusted and adjusted associations between individual predictors and care-seeking through univariate and multivariable logistic regression models, respectively. We accounted for correlation between repeated observations from the same child through the use of generalized estimating equations [35], specifying the binomial family, logit link, and an exchangeable correlation structure with robust estimation of standard errors. Variables were selected based on their previously demonstrated association with care-seeking. All variables were included in the final model except the presence of danger signs, which was excluded due to collinearity with illness severity and multiple reported symptoms. We also assessed for potential nonlinear associations and effect modification, including an interaction term in the final model between illness severity and maternal employment status. Records with missing data (20%) were excluded from analysis according to list-wise deletion with the distribution of missing data by variable as follows: severity (14%), household structure (3%), maternal employment (2%), health insurance coverage (2%), and number of symptoms (<1%). Sensitivity analysis considered the influence of missing data through multiple imputation of missing values. Missing values were estimated according to univariate imputation models including care-seeking status and all other relevant predictors without missing data (eg, study group, participant ID, child age, etc.). All statistical analyses were conducted in Stata 14 (Stata Corp, College Station, TX, USA) [36].

In addition to evaluating determinants of care-seeking, we also explored sequential patterns of care-seeking. Mothers who reported any care-seeking during an episode of childhood illness were asked to list each source from which care was sought, including the name, type, and number of days ago when care was sought. These data were linked to form sequences of care sought during an episode of childhood illness, starting with the first provider where care was sought and continuing with additional providers. Individual provider types were recoded into four major response categories to facilitate identification of common patterns: public facility, public non-facility, private facility and pharmacy. Care-seeking sequences were further stratified according to illness severity.

### Ethical approval

Written consent was provided by all participants prior to enrollment. The study protocol was approved by the ethics committees of the University of Edinburgh and K.E.M. Hospital Research Centre, Pune (Study ID No. 1415).

## RESULTS

### Descriptive analyses

Field workers enrolled 1066 children ages 0-4 years from 743 households between May and June 2015 (**Table 1**). Mean child age at enrollment was 33 months (standard deviation (SD) = 5) with 52% male children (n = 551). Mothers had a mean age at enrollment of 25.3 years (SD = 3.3) with two thirds completing 10 or more years of schooling and a quarter reporting being currently employed. Mothers most frequently reported a single child under five (59%), followed by two (38%) and three children (3%), respectively.

**Table 1:** Baseline child and household characteristics, May-June 2015, rural Pune district, India

Characteristic	N (%)
**Child Characteristics (n = 1066)**	
Child age, months, mean (SD)	33 (15)
Child sex:	
Male	551 (52)
Female	515 (48)
**Household characteristics (n = 759)**	
Maternal age, years, mean (SD)	25.3 (3.3)
Maternal education, years completed:	
0-7	111 (15)
8-9	114 (15)
10-11	212 (29)
12+	306 (41)
Mother currently employed:	206 (28)
Number of children <5 y per household:	
One	442 (59)
Two	279 (38)
Three	22 (3)
Religion of household head:	
Hindu	678 (91)
Muslim	23 (3)
Buddhist or Neo-Buddhist	34 (5)
Other	8 (1)
Family structure:	
Extended	365 (51)
Nuclear	348 (49)
Insurance ownership:	135 (19)
Urban residence:	506 (68)
Distance to nearest health facility (public or private), km:	
<1	426 (57)
1-3	251 (34)
>3	66 (9)
Nearest facility by sector:	
Public	103 (14)
Private	640 (86)

km – kilometer, SD – standard deviation

**Figure 1:**
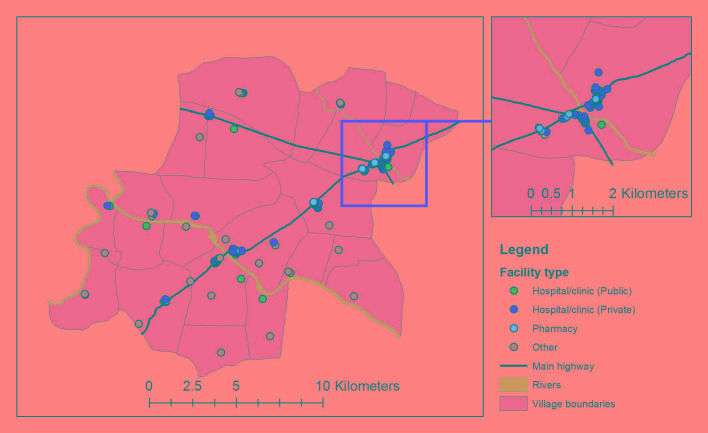
Study area of the Vadu Health and Demographic Surveillance System with all facilities identified during health facility census, February to May 2015, rural Pune district, India. Note: Public sector hospitals and clinics include the rural hospital (1), primary health center (1), and sub-centers (7). Private sector hospitals and clinics include private hospitals (49), private clinics (43), and the non-governmental organization/trust hospital (1). Pharmacies include only pharmacies/drugstores (68). Other facilities include Anganwadi/Integrated Child Development Services centers (24) and shops (4).

Households were primarily Hindu religion (91%) with a similar proportion reporting extended and nuclear household structure and one fifth of households reporting some health insurance coverage. Two thirds of participant households were located in urban villages with 57% of participants less than one kilometer from the nearest health facility and 34% of participants between one and three kilometers from the nearest health facility (**Figure 1**). The nearest provider type was most often a private hospital or clinic.

**Figure 2:**
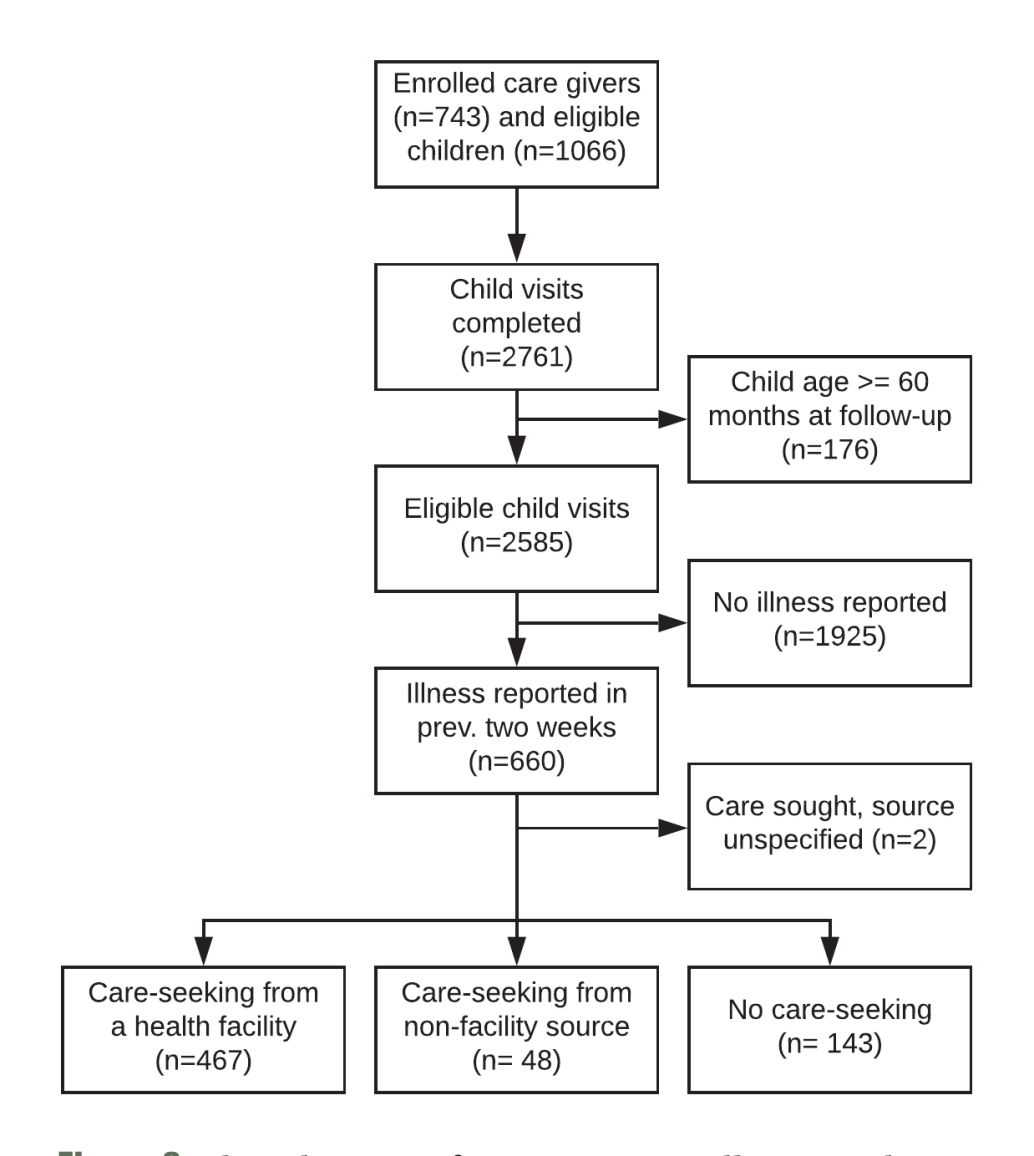
Flow diagram of participant enrollment and follow-up.

Field workers completed 1993 household visits between July 2015 and February 2016 (90% completion), administering the follow-up questionnaire to caregivers of 2761 children (**Figure 2**). Of these records, 176 (6%) were excluded as the child had completed his or her fifth birthday before follow-up. Mothers reported one or more symptom of childhood illness at 660 of the remaining 2585 records (26%).

**Figure 3:**
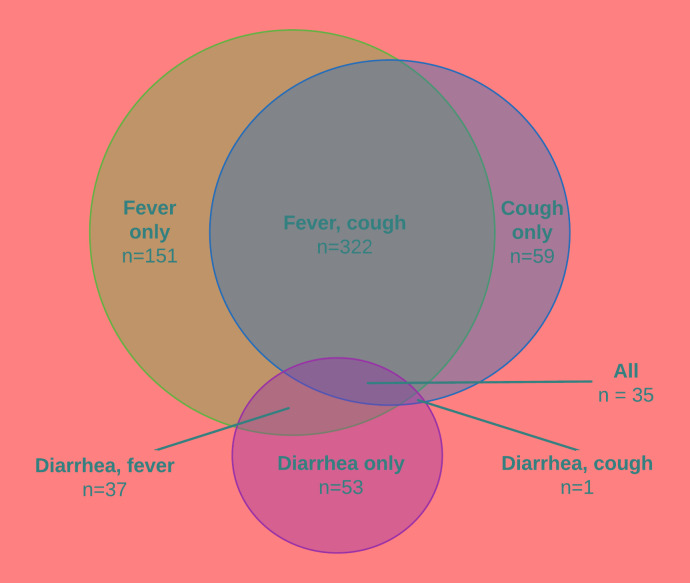
Illness profile of 658 children reporting one or more symptoms of childhood illness in the previous two weeks between July 2015 to February 2016 in rural Pune district, India.

Completed care-seeking data were reported for 658 (99%) of these records, forming the sample for the remaining analyses. Fever was reported in 83% of instances of child illness, followed by cough (64%) and diarrhea (19%) (**Figure 3**, Table S1 in the **Online Supplementary Document**). Childhood illnesses were frequently multi-symptomatic (60%) with combined fever and cough accounting for half of all reported illness episodes.

**Table 2** presents care-seeking for childhood illnesses stratified by maternally reported illness severity. Care was sought from a health facility during 71% of illness episodes overall, increasing from 47% for non-severe illness to 88% for somewhat severe illness and 100% for very severe illness. Care was primarily sought from the private sector across all severity strata with all episodes of very severe illness seeking care exclusively from the private sector.

**Table 2:** Care-seeking by illness severity for 658 childhood illness episodes reported between July 2015 and February 2016, rural Pune district, India

Care seeking	All cases N (%)	Non-severe N (%)	Moderately severe N (%)	Very severe, N (%)	Severity missing N (%)
No care sought	143 (22)	96 (40)	22 (7)	0 (0)	25 (27)
Care sought from any source	515 (78)	142 (60)	272 (93)	32 (100)	69 (73)
Care sought, health facility:	467 (71)	113 (47)	258 (88)	32 (100)	64 (68)
-Public sector facility*	35 (5)	7 (3)	22 (7)	0 (0)	6 (6)
-Private sector facility†	438 (67)	106 (45)	242 (82)	32 (100)	58 (62)
-Care sought, non-facility source	48 (7)	29 (13)	14 (5)	0 (0)	5 (5)
Total	658 (100)	238 (100)	294 (100)	32 (100)	94 (100)

*Sum of care sought from public and private sector facilities may exceed care sought from health facility due to multiple reported care-seeking events per illness episode.

†Public sector facility sources include: rural hospital, primary health center, and sub-centre/auxiliary nurse midwife. Private health facility sources include: private hospital, private doctor/clinic, and non-governmental organization or trust hospital/clinic.

### Care-seeking determinants

**Table 3** presents descriptive characteristics of 467 cases where a child was brought for care and 191 cases where a child was not brought for care. Mothers of children brought for care were more likely to report somewhat or very severe illness (*P* < 0.001), more than one symptom (*P* < 0.001), and the presence of one or more danger signs (*P* = 0.005). Maternal employment was less common among cases for which a child was brought for care (*P* = 0.03).

**Table 3:** Descriptive characteristics of participants seeking care and not seeking care for childhood illness from July 2015 to February 2016, rural Pune district, India*

Characteristic	Care sought (N = 467), N (%)	Care not sought (N = 191), N (%)	*P*-value
**Child characteristics**			
Child age, months, mean (SD):	33.4 (14.5)	32.9 (14)	0.66
Child sex:			
Male	234 (50)	95 (50)	0.93
Female	233 (50)	96 (50)	
Perceived severity:			
Very severe	32 (8)	0 (0)	<0.001
Moderately severe	258 (64)	36 (22)	
Non-severe	113 (28)	125 (78)	
Number of reported symptoms:			
Single	148 (32)	113 (60)	<0.001
Multiple	318 (68)	77 (40)	
Reported danger signs:			
No	402 (89)	168 (96)	0.005
Yes	51 (11)	7 (4)	
**Maternal characteristics**			
Maternal education, completed years:			
0-7	65 (14)	34 (18)	0.25
8-9	71 (15)	19 (10)	
10-11	142 (30)	60 (31)	
12+	189 (41)	78 (41)	
Maternal employment status:			
Not currently employed	358 (78)	128 (70)	0.03
Currently employed	102 (22)	56 (30)	
**Household characteristics**			
Number of children under-five in household:			
Single	222 (48)	79 (41)	0.15
Multiple	245 (52)	112 (59)	
Household structure:			
Extended	244 (54)	90 (49)	0.33
Nuclear	210 (46)	92 (51)	
Religion of head of household:			
Hindu	417 (89)	165 (86)	0.29
Other	50 (11)	26 (14)	
**Wealth quintile**			
Quintile 1 (lowest)	92 (20)	46 (24)	0.62
Quintile 2	69 (15)	26 (14)	
Quintile 3	102 (22)	38 (20)	
Quintile 4	110 (24)	49 (26)	
Quintile 5 (highest)	94 (20)	32 (17)	
**Health insurance coverage**			
No	355 (77)	155 (83)	0.11
Yes	105 (22)	32 (17)	
**Residence**			
Rural	162 (35)	64 (34)	0.77
Urban	305 (65)	127 (66)	
**Distance to nearest health facility**			
<1 km	280 (60)	115 (60)	0.68
1-3 km	146 (31)	63 (33)	
>3 km	41 (9)	13 (7)	

SD – standard deviation

*Difference in child’s age at follow-up calculated using Student’s T-Test. Differences for all other variables calculated using Pearson χ-2 test. Tests are not clustered by participant.

**Table 4** presents the results of unadjusted and adjusted logistic regression analyses of care-seeking from a health facility. The adjusted analysis includes the interaction between illness severity and maternal employment status, strata specific estimates of which are presented in **Table 5**. Children with multiple symptoms were more than twice as likely to be brought for care as children with a single symptom (odds ratio (OR) = 2.4, 95% CI = 1.5-3.9). Children with somewhat severe and very severe illness were seven times more likely to seek care than children with non-severe illness in households where the mother was not employed (OR = 7.0, 95% CI = 3.9-12.6) and five times more likely to seek care in households where the mother was employed (OR = 5.0, 95% CI = 2.2-11.2). Maternal employment was associated with a significant decrease in care-seeking among cases of non-severe illness (OR = 0.3, 95% CI = 0.1-0.7) but not significantly associated with care-seeking among somewhat severe and very severe cases. Children from a household where one or more member was covered by health insurance were more than twice as likely to be brought to a health facility for care (OR = 2.2, 95% CI = 1.1-4.3). Child age, child sex, maternal education, household socioeconomic status, urban residence, and proximity to a health facility were not associated with care seeking in either unadjusted or adjusted analyses. Sensitivity analysis applying multiple imputation detected no significant differences in coefficient estimates or confidence intervals for number of reported symptoms, perceived illness severity, maternal employment, or its interaction with perceived severity Tables S2-S3 and Figure S2 in the **Online Supplementary Document**). A minor difference was observed in the estimated effect of health insurance coverage, resulting in this variable being marginally significant within the multiply imputed analysis (OR = 1.8, 95% CI = 1.0-3.3).

**Table 4:** Univariate and multivariable logistic regression results of care-seeking for childhood illness between July 2015 to February 2016, rural Pune district, India*

Characteristic	Unadjusted OR (95% CI)	Adjusted OR (95% CI)
Multiple symptoms reported	3.2 (2.2-4.6)^†^	2.4 (1.5-3.9)^†^
Presence of danger signs	3.1 (1.4-6.9)^†^	See note
Illness perceived as moderate-to-very severe	8.8 (5.5-13.9)^†^	7.0 (3.9-12.6)^†^
Mother currently employed	0.6 (0.4-0.9)^‡^	0.3 (0.1-0.7)^†^
Interaction: severity × maternal employment	N/A	2.3 (0.8-6.8)
Maternal education, completed years:		
0-7	REF	REF
8-9	1.7 (0.9-3.3)	1.4 (0.5-3.7)
10-11	1.2 (0.7-2.1)	1.0 (0.5-2.2)
12+	1.2 (0.7-2.0)	0.8 (0.4-1.8)
Child age, months	1.0 (1.0-1.0)	1.0 (1.0-1.0)
Child being female	1.0 (0.7-1.5)	1.0 (0.6-1.7)
Other children under-five in household	0.8 (0.6-1.2)	0.8 (0.5-1.2)
Household structure		
Extended	REF	REF
Nuclear	0.8 (0.6-1.2)	0.7 (0.4-1.4)
Religion of head of household:		
Hindu	REF	REF
Other	0.8 (0.5-1.3)	0.8 (0.4-1.6)
Wealth quintile:		
Quintile 1 (lowest)	REF	REF
Quintile 2	1.3 (0.7-2.4)	1.2 (0.5-2.8)
Quintile 3	1.3 (0.7-2.3)	1.4 (0.6-3.1)
Quintile 4	1.1 (0.6-1.8)	0.9 (0.4-2.1)
Quintile 5 (highest)	1.4 (0.8-2.5)	1.8 (0.6-4.8)
Covered by health scheme or health insurance	1.4 (0.9-2.3)	2.2 (1.1-4.3)^‡^
Urban residence	1.0 (0.7-1.5)	1.2 (0.6-2.3)
Distance to nearest health facility:		
<1 km	REF	REF
1-3 km	1.0 (0.6-1.4)	1.1 (0.6-2)
>3 km	1.4 (0.7-2.7)	0.9 (0.3-2.5)

OR – odds ratio, CI – confidence interval, REF – reference group, km – kilometer

*Presence of danger signs excluded from final model due to collinearity with other covariates. Confidence intervals estimated using robust standard errors.

^†^*P* < 0.01.

^‡^*P* < 0.05.

**Table 5:** Interaction between illness severity and maternal employment on care-seeking for childhood illness between July 2015 and February 2016, rural Pune district, India*

	Illness severity		
	**Non-severe, OR (95% CI)**	**Moderate-to-very severe, OR (95% CI)**	**Effect of severity within employment strata, OR (95% CI)**
**Maternal employment:**			
Not currently employed	1.0 (Reference)	7.0 (3.9-12.6)^†^	7.0 (3.9-12.6)^†^
Currently employed	0.3 (0.1-0.7)^†^	5.0 (2.3–11.2)^†^	16.4 (6.7-40.2)^†^
Effect of employment status within severity strata	0.3 (0.1-0.7)^†^	0.7 (0.3-1.6)	

OR – odds ratio; CI – confidence interval

*Measure of interaction on multiplicative scale: ratio of ORs (95% CI) = 2.3 (0.8-6.8). ORs are adjusted for number of reported symptoms, maternal education, child age, child sex, household structure, religion of head of household, household SES, health insurance coverage, urban residence, and distance to nearest health facility. Confidence intervals estimated using robust standard errors.

^†^*P* < 0.01.

### Care-seeking patterns

A supplementary module on care-seeking steps was completed for 468 (91%) cases where care was sought from any source, including facility and non-facility sources. These data were linked to form sequences of care-seeking patterns, presented across all reported episodes and stratified by illness severity (**Table 6**). The most common care-seeking pattern overall and within each illness stratum was a single visit to a private provider, accounting for two thirds of care-seeking for non-severe cases and increasing to 79% among very severe cases. The second most common pattern overall and among somewhat severe and very severe cases was care-seeking from two or more private sector providers, while the second most common pattern for non-severe illness was care-seeking exclusively from pharmacies and drugstores (18%). As illness severity increases, the proportion of care-seeking only from pharmacies or non-facility public sector sources decreases substantially, accounting for 21% of care-seeking for non-severe illness, 4% for somewhat severe illness, and no care-seeking for very severe illness. We observed minimal crossover between sectors with only 1% of children (n = 4) beginning in the private sector and less than 1% of children (n = 2) beginning in the public sector brought for follow-up care in the opposite sector, respectively.

**Table 6:** Sequential care-seeking patterns by illness severity for 468 illness episodes reporting any care-seeking and completing supplemental questionnaire between July 2015 and February 2016, rural Pune district, India

Care seeking sequence	All cases N (%)	Non-severe N (%)	Moderately severe N (%)	Very severe N (%)	Severity missing N (%)
Pvt. Fac. only	334 (71)	89 (67)	186 (73)	22 (79)	37 (70)
Pvt. Fac.→Pvt. Fac.*	51 (11)	8 (6)	30 (12)	6 (21)	7 (13)
Pharm. †, ‡ only	38 (8)	24 (18)	10 (4)	-	4 (8)
Pub. Fac. only	29 (6)	7 (5)	17 (7)	-	5 (9)
Pharm.→Pvt. Fac.	5 (1)	1 (1)	4 (2)	-	-
Other Pub.3 only	5 (1)	4 (3)	1 (<1)	-	-
Pvt. Fac.→Pub. Fac.	4 (1)	-	4 (2)	-	-
Pub. Fac.→Pvt. Fac.	2 (<1)	-	2 (1)	-	-
Pharm.→Pvt. Fac.→Pharm.	1 (<1)	-	1 (<1)	-	-
Total	468 (100)	133 (100)	254 (100)	28 (100)	53 (100)

Pvt. Fac. – Private sector facility, including private hospital, private doctor/clinic, and non-governmental organization or trust hospital/clinic, Pharm. – Pharmacy/drugstore, Pub. Fac. – Public sector facility, including rural hospital, primary health center, and sub-centre/auxiliary nurse midwife, Other Pub. – Non-facility public sector source, including Anganwadi/Integrated Child Development Center and Accredited Social Health Activist workers

*Includes eight records with three or more care-seeking events exclusively from private facilities.

†Includes two records with two care-seeking events exclusively at pharmacies.

‡Care sought only from these non-facility sources is excluded when calculating care seeking from a health facility.

## DISCUSSION

Care-seeking for childhood illness in India has increased in the last decade both at the national level and within Maharashtra, where state-level data report care-seeking for diarrhea, fever, and symptoms of ARI during 78%, 85%, and 89% of episodes, respectively. Achieving further improvement in care-seeking within the state and nationally requires understanding the determinants and patterns of care-seeking for childhood illness. Through a population-based cohort of 1066 children under-five in rural Pune district, India, we observed high levels of care-seeking overall with illness characteristics as the key determinants of care-seeking. Children whose illnesses were reported as somewhat-to-very severe were seven times more likely to be brought for care than children whose illness was non-severe and children whose mothers reported they had more than one symptom were more than twice as likely to be brought for care.

One tenth of care-seeking involved self-medication through private pharmacies, typically as the only source of care though occasionally followed by facility-based care. While pharmacies may provide care for mild childhood illness (eg, oral rehydration therapy for diarrhea), the quality of services they provide is often low and has been linked with medication misuse [37-39]. A review of pharmacy performance in Asian countries observed poor adherence to guidelines for the treatment of diarrhea with pharmacists recommending oral rehydration solution less than half of the time while frequently providing antibiotics and other unnecessary medicines [40]. Within our study, the pattern of pharmacy-based care varied with perceived illness severity. Nearly 20% of children with non-severe illness were brought to pharmacies and drugstores for care compared to 6% of children with somewhat severe illness and no children with very severe illness. Decreased care-seeking from pharmacies among more severe illness is consistent with a recent review of medication misuse in India, reporting that self-medication was preferred among mild illness as a strategy to avoid the cost of doctor consultation or diagnostic tests [39]. Similarly, mild perceived severity was associated with the decision to seek care directly from a pharmacy among adults with symptoms of ARI in Bangladesh [41].

No association was observed between child sex and care-seeking for childhood illness. This is consistent with the most recent care-seeking estimates for Maharashtra, where similar proportions of facility-based care-seeking were observed by child sex for diarrhea, fever, and symptoms of ARI [5]. Similar care-seeking by child sex has also been reported elsewhere in India [42]. While sex did not appear to be associated with the decision to seek care, this does not exclude potential bias at other stages of the management of childhood illness. Previous findings from the study area identified male preference in utilization of private providers, distance traveled for care, money spent, and compliance with referral [21]. A study in Uttar Pradesh found no association between child sex and care-seeking for neonatal illness but noted both delays in illness recognition and significantly less money spent on girls [43]. Exploring sex bias across all stages in the management of childhood illness was beyond the scope of the study, which restricted its focus to facility-based care-seeking. Further research is needed to assess for bias across the other steps in the pathway from illness recognition to type of care sought and compliance with referral.

Children with non-severe illness were 70% less likely to be brought for care if their mother was currently employed, while maternal employment was not significantly associated with care-seeking for somewhat-to-very severe illness. Previous studies exploring the role of maternal employment in health utilization have found mixed results. An analysis of national data in India linked improved child immunization status and care-seeking practices with high maternal autonomy, measured as financial access, freedom of movement, and decision-making power [44]. Sometimes used as a proxy for maternal autonomy, maternal employment was included as a separate covariate in the analysis and was found to be associated with both poorer immunization status and decreased care-seeking for childhood illness [44]. In contrast, maternal employment was associated with increased care-seeking for obstetric complications among women in Bangladesh [45]. Within our study context, the interaction between employment and illness severity may indicate that the opportunity cost of seeking care for employed mothers is sufficient to result in lower care-seeking for non-severe illness but insufficient to deter mothers from seeking care for more severe illness.

Maternal education, access to care, and SES have previously been found to be associated with care-seeking for childhood illness but did not appear significant within our study. This should be interpreted alongside the baseline distributions of these characteristics within the study population. Mothers in our study area are highly educated with three quarters completing 10 or more years of schooling, compared to one quarter of similarly aged women in Maharashtra [5]. Household access to care was also high with more than half of households located within one kilometer of health facility and most remaining households located less than three kilometers from a health facility. While not explicit measurements of household SES, high levels of education and access to care would suggest higher than average SES within the study population relative to state and national estimates. As underlying SES of the study population increases, the increment between calculated SES quintiles becomes less meaningful. For example, the lowest and highest quintiles in our sample may still represent the poorest and wealthiest households, respectively, but if the poorest are still relatively wealthy then we would not expect to see much difference between groups with regard to care-seeking or other variables typically associated with SES. Our null findings with regard to these variables may therefore rule out the potential for a large effect size but may be consistent with a smaller one.

This study includes limitations. First, data on childhood illness and related care-seeking behavior during the previous two weeks were collected during participant interviews and may be subject to a combination of recall and social desirability bias. A recent study of the validity of maternally-reported care-seeking for childhood illness found that the indicator had both high sensitivity and specificity, resulting in only a minor overestimation of care-seeking relative to true levels [46]. Previous experience within this study area noted the potential for underreporting of sensitive health behaviors (eg, abortion services), especially when solicited through a survey-based approach [47], though this bias is likely reduced when asking about less sensitive behaviors. Second, the high levels of sociodemographic indicators (eg, mother’s education, access to care) observed within our study population limit the generalizability of our findings to the state of Maharashtra and similar contexts [5]. The study area represents a community in transition, historically based in agriculture but subject to the urbanizing influence of the nearby metropolitan area. As large urban centers continue to expand throughout India and elsewhere, the number of such communities will continue to grow. Third, distance to the nearest health facility was estimated as the shortest path along the road network and did not account for other factors potentially affecting travel time, such as road type or the transportation mode used, as the inputs required for such an approach were unavailable in this study. Furthermore, a comparison of methods to calculate geographic access to care found similar results across methods of varying levels of sophistication, suggesting that a less sophisticated approach may provide a reasonable measurement of geographic access in the context of low- and middle-income countries [48]. Finally, one fifth of illness records included at least one variable with a missing response with illness severity missing most frequently. Excluding these records from analysis may have reduced statistical efficiency and yielded null results where a significant association might otherwise be detected. Similarly, excluding records with missing values may have resulted in biased estimates if these data were systematically different from those that were not missing. Our sensitivity analysis comparing completed cases alone with the multiply imputed data set yielded similar results, suggesting no significant bias in our results due to missing data.

## CONCLUSIONS

This study demonstrates the importance of maternal perception of illness severity in determining facility-based care-seeking for childhood illness in rural Pune district, India. A further association between maternal employment and decreased care-seeking was noted in non-severe cases, though not in somewhat-to-very severe cases. Additional research is required to determine whether male bias exists at different stages in the management of childhood illness, such as illness recognition and resources allocated to treatment. As care-seeking for childhood illness continues to rise in India, there is an urgent need to develop indicators to assess the content of care, especially with regard to the appropriateness of treatments for the illness being treated [49].

## Additional material

Online Supplementary Document
