# Supplementary material for: Determinants and patterns of care-seeking for childhood illness in rural Pune District, India
Source: J Glob Health. 2020 Jan 31;10(1):010601. doi: 10.7189/jogh.10.010601 (PMC7020658; doi:10.7189/jogh.10.010601)

Appendix I: Appendix Tables and Figures

Figure S1: Participant Follow-up Schedule

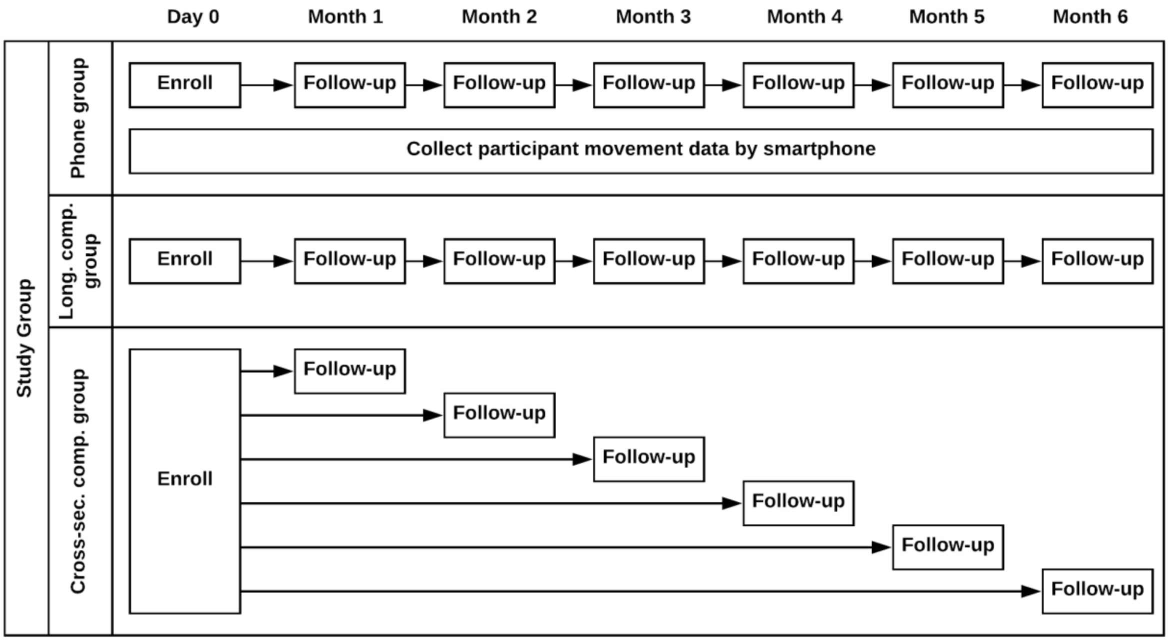

Cross-sec. comp. group - Cross-sectional Comparison Group; Long. comp. group - Longitudinal comparison group. Reproduced with permission from Hirve et al. (2018).

Table S1. Illness characteristics of 658 childhood illness episodes reported between July 2015 and February 2016, rural Pune district, India

Characteristic	N (%)
Single symptom	263 (40)
Diarrhea only	53 (8)
Fever only	151 (23)
Cough only	59 (9)
Suspected ARI	3 (0)
Multiple symptoms	395 (60)
Diarrhea, fever	37 (6)
Diarrhea, cough	1 (0)
Fever, cough	322 (49)
Fever, Suspected ARI	30 (5)
Diarrhea, fever, cough	35 (5)
Diarrhea, fever, Suspected ARI	7 (1)
Presence of danger signs	63 (10)
Vomiting	41 (7)
Difficulty eating	12 (2)
Child unusually sleepy/unconscious	5 (1)
Lower chest in-drawing	5 (1)
Convulsions	0 (0)
Perceived severity	
Non-severe	32 (5)
Moderately severe	294 (47)
Very severe	238 (38)
Missing	94 (14)
Status at follow-up interview	
Resolved	403 (64)
Not resolved	225 (36)
Symptom onset, mean days prior to interview (SD)	
Resolved episodes	9 (4)
Unresolved episodes	5 (4)
Illness duration*, mean days (SD)	4 (2)

* Illness duration calculated for resolved episodes only

Table S2. Multiply imputed univariate and multivariable logistic regression results of care-seeking for childhood illness between July 2015 to February 2016, rural Pune district, India

Characteristic	Unadjusted OR (95% CI)	Adjusted OR (95% CI)
Multiple symptoms reported	3.2 (2.2 - 4.5)†	2.5 (1.7 - 3.9)†
Presence of danger signs	3.1 (1.4 - 7.1)†	See note
Illness perceived as moderate-to-very severe	8.6 (5.5 - 13.4)†	7.3 (4.2 - 12.9)†
Mother currently employed	0.6 (0.4 - 0.9)‡	0.3 (0.2 - 0.7)†
Interaction: severity x maternal employment	N/A	2.0 (0.7 - 5.6)
Maternal education, completed years		
0-7	REF	REF
8-9	1.7 (0.9 - 3.3)	1.7 (0.7 - 4.1)
10-11	1.2 (0.7 - 2.1)	1.1 (0.6 - 2.3)
12+	1.2 (0.7 - 2)	1.0 (0.5 - 2.1)
Child age, months	1.0 (1.0 – 1.0)	1.0 (1.0 – 1.0)
Child being female	1.0 (0.7 - 1.5)	1.1 (0.7 - 1.7)
Other children under-five in household	0.8 (0.6 - 1.2)	0.8 (0.5 - 1.2)
Household structure		
Extended	REF	REF
Nuclear	0.8 (0.6 - 1.2)	0.7 (0.4 - 1.3)
Religion of head of household		
Hindu	REF	REF
Other	0.8 (0.5 - 1.3)	0.8 (0.4 - 1.5)
Wealth quintile		
Quintile 1 (lowest)	REF	REF
Quintile 2	1.3 (0.7 - 2.5)	1.2 (0.6 - 2.4)
Quintile 3	1.3 (0.7 - 2.3)	1.6 (0.8 - 3.3)
Quintile 4	1.1 (0.6 - 1.9)	1.0 (0.4 – 2.0)
Quintile 5 (highest)	1.4 (0.8 - 2.5)	1.7 (0.7 - 4.2)
Covered by health scheme or health insurance	1.4 (0.9 - 2.3)	1.8 (1.0 - 3.3)§
Urban residence	1.0 (0.7 - 1.5)	1.3 (0.7 - 2.4)
Distance to nearest health facility		
<1 km	REF	REF
1-3 km	1.0 (0.6 - 1.4)	1.3 (0.8 - 2.2)
>3km	1.4 (0.7 - 2.7)	1.5 (0.6 - 3.7)

CI - confidence interval; REF - reference group; km - kilometer

***** Presence of danger signs excluded from final model due to collinearity with other covariates. Confidence intervals estimated using robust standard errors.

† *P*<0.01.

‡ *P*<0.05.

§ *P*<0.10.

Table S3. Multiply imputed results for interaction between illness severity and maternal employment on care-seeking for childhood illness between July 2015 and February 2016, rural Pune district, India

	Illness severity		
	Non-severe<br>OR (95% CI)	Moderate-to-very severe<br>OR (95% CI)	*Effect of severity within employment strata*<br>OR (95% CI)
**Maternal employment**			
Not currently employed	1.0<br>(Reference)	7.3<br>(4.2 - 12.9)†	7.3<br>(4.2 - 12.9)†
Currently employed	0.3<br>(0.2 - 0.7)†	4.6<br>(2.1 - 9.8)†	14.3<br>(5.9 - 34.6)†
Effect of employment status within severity strata	0.3<br>(0.2 - 0.7)†	0.6<br>(0.3 - 1.4)	

OR - odds ratio; CI - confidence interval

* Measure of interaction on multiplicative scale: ratio of ORs (95% CI) = 2.0 (0.7 - 5.6). ORs are adjusted for number of reported symptoms, maternal education, child age, child sex, household structure, religion of head of household, household SES, health insurance coverage, urban residence, and distance to nearest health facility. Confidence intervals estimated using robust standard errors.

† P<0.01.

Figure S2: Comparison of selected regression results from multivariable model without imputation and with imputation

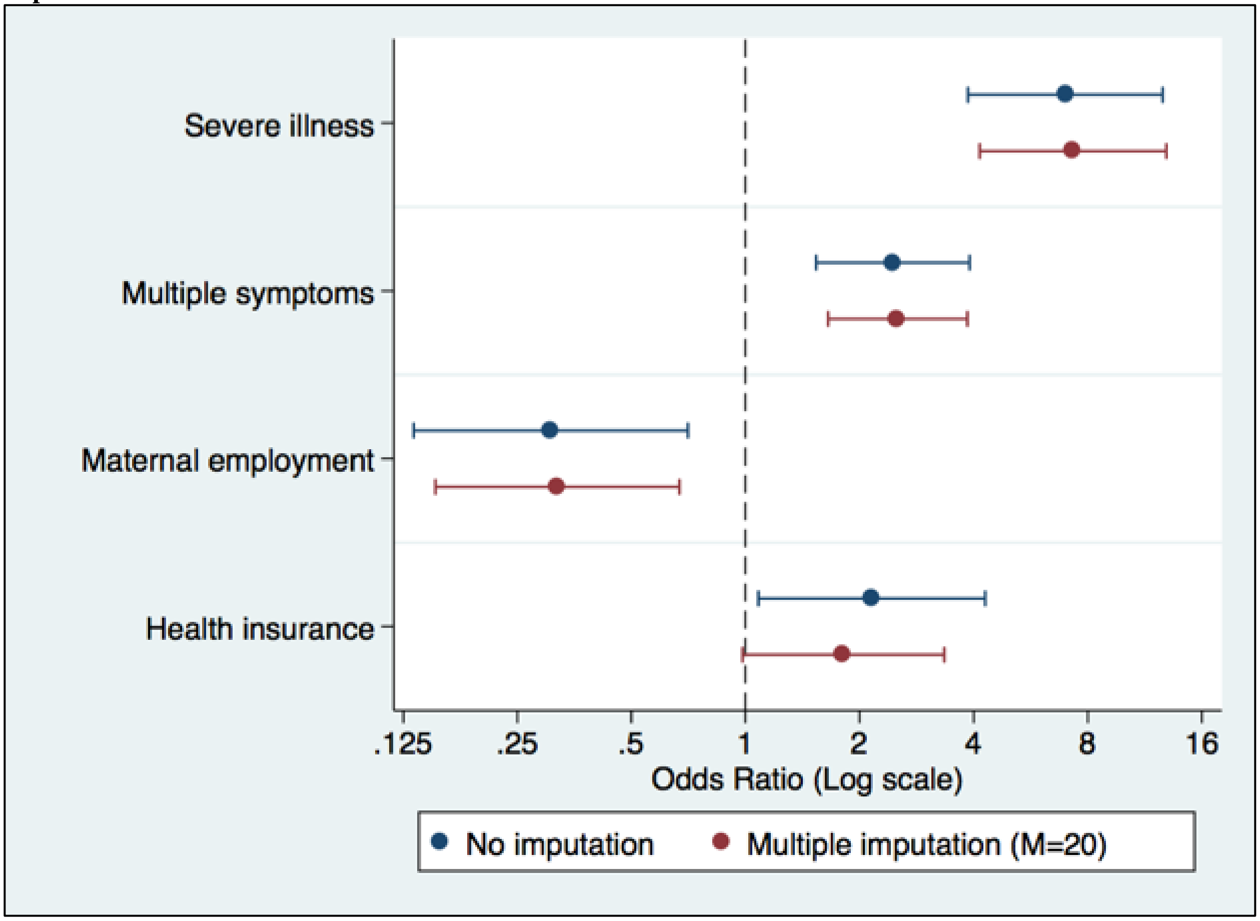

Supplement: Online Supplementary Document [file jogh-10-010601-s001.pdf]
